# The Contribution of Advanced Glycation End product (AGE) accumulation to the decline in motor function

**DOI:** 10.1186/s11556-016-0163-1

**Published:** 2016-03-04

**Authors:** Hans Drenth, Sytse Zuidema, Steven Bunt, Ivan Bautmans, Cees van der Schans, Hans Hobbelen

**Affiliations:** Research Group Healthy Ageing, Allied Healthcare and Nursing, Hanze University of Applied Sciences, PO Box 3109, 9701 DC Groningen, The Netherlands; Zuid Oost Zorg, Organisation for Elderly Care. Burg, Wuiteweg 140, 9203 KP Drachten, The Netherlands; Department of General Practice, University of Groningen, University Medical Center Groningen, PO Box 196 9700 AD, Groningen, HPC FA21 The Netherlands; Frailty in Ageing Research Group and Gerontology Department, Free University of Brussels, Laarbeeklaan 103, B-1090 Brussels, Belgium

**Keywords:** Advanced Glycation End products, Motor function, Ageing, Biomarker, Systematic review

## Abstract

Diminishing motor function is commonly observed in the elderly population and is associated with a wide range of adverse health consequences. Advanced Glycation End products (AGE’s) may contribute to age-related decline in the function of cells and tissues in normal ageing. Although the negative effect of AGE’s on the biomechanical properties of musculoskeletal tissues and the central nervous system have been previously described, the evidence regarding the effect on motor function is fragmented, and a systematic review on this topic is lacking. Therefore, a systematic review was conducted from a total of eight studies describing AGE’s related to physical functioning, physical performance, and musculoskeletal outcome which reveals a positive association between high AGE’s levels and declined walking abilities, inferior ADL, decreased muscle properties (strength, power and mass) and increased physical frailty. Elevated AGE’s levels might be an indication to initiate (early) treatment such as dietary advice, muscle strengthening exercises, and functional training to maintain physical functions. Further longitudinal observational and controlled trial studies are necessary to investigate a causal relationship, and to what extent, high AGE’s levels are a contributing risk factor and potential biomarker for a decline in motor function as a component of the ageing process.

## Background

In the ageing population, decline in motor function such as reduced walking speed, poor balance, and loss of muscle strength are commonly observed phenomena [1–4]. Most human physiologic systems regress with ageing, independently of substantial disease effects, at an average linear loss rate of 0.34–1.28 % per year between the age of 30 and 70 years [5]. The age related loss of skeletal muscle mass, which is accompanied by muscle weakness, is an important contributor to functional decline [6]. More people over the age of 70 are having difficulties performing everyday functions because of motor function related problems [7]. Impaired motor function is a prominent characteristic of physical frailty and is associated with a wide range of adverse health consequences such as falls, disability, death, hospitalization, and institutionalization [8, 9]. In addition to physical frailty, longitudinal studies suggest that a decreased level of motor function is associated with a decline of cognitive function and a subsequent development of both mild cognitive impairment and Alzheimer’s disease [3]. A decline in motor function in older ages could be predicted by biomarkers which could allow for early interventions. Since the early 1980’s, it is proposed that the cross-linking of long-lived proteins mediated by Advanced Glycation End products (AGE’s) may contribute to the age-related decline of the functioning of cells and tissues in normal ageing. AGE’s could, therefore, be a potential biomarker and risk factor for the decline of motor function in the older population [10].

### Advanced glycation End products and ageing

The occurrence of AGE’s is mediated by non-enzymatic condensation of a reducing sugar with an amino group. AGE’s are spontaneously produced in human tissues as an element of normal metabolism which increases with aging [11–14]. The increase of the level of free/unbound and protein bound AGE’s in the blood circulation is also determined by an exogenous intake such as food [15]. AGE’s are being removed from the body through enzymatic clearance and renal excretion.

It has been proposed that, with ageing, there is an imbalance between the formation and natural clearance of AGE’s, leading to the accumulation of AGE’s in tissues [16, 17]. In many age related diseases, the accumulation of AGE’s is a significant contributing factor in degenerative processes, especially in renal failure, blindness, cardiovascular diseases, and complications of diabetes mellitus [13, 18]. Elevated AGE’s levels in patients with diabetes mellitus (DM) is most likely due to an excessive elevation of glucose concentration which consequently accelerates the glycation of proteins [11, 13, 18]. Furthermore, AGE’s play a role in neurological disorders such as Alzheimer’s Disease (AD) [13, 19]. Interestingly, Hobbelen et al. [20] ascertained that patients in early stage of dementia and with DM had a significantly higher risk for the development of muscle stiffness/hypertonia (defined as paratonia) compared to those with dementia but without DM (OR = 10.7, 95 % CI:2.2–51.7). In early stage dementia, paratonia already negatively and significantly impacts functional mobility such as walking speed. As the most common cause of dementia, Alzheimer’s disease, as well as DM, are related to higher serum concentrations of AGE’s, the previous finding in the Hobbelen et al. study prompted the hypothesis that AGE’s may partly or indirect be responsible for the development of paratonia. The pathogenesis of paratonia is unclear and central nervous system changes as well as peripheral biomechanical changes are hypothesized [20].

Several types of AGE’s have been described and can be categorised into fluorescent cross-linking AGE’s, non-fluorescent cross-linking AGE’s, and non-crosslinking AGE’s. The best chemically characterized AGE’s are Pentosidine (fluorescent cross-linking) and Carboximethyl-lysine (CML, non-cross-linking) [21]. Cross-linking AGE’s are responsible for an increasing proportion of insoluble extracellular matrix and thickening of tissue as well as increasing mechanical stiffness and loss of elasticity [12, 22, 23]. Cross-linking AGE’s are considered as being involved in the pathophysiology of arthritis [24, 25]. In fact, the accumulation of AGE’s in human articular cartilage increases cartilage stiffness and brittleness. Consequently, the cartilage becomes increasingly prone to damage and degeneration [26, 27]. In rheumatoid arthritis, elevated levels of cross-linking AGE’s in serum and/or synovial fluids also appear to correlate with the levels of inflammatory markers and the disease activity [24]. Another impact on the musculoskeletal system is within the skeletal bone where AGE-induced crosslinks in the collagen matrix alter the mechanical properties of bone by increasing stiffness and fragility [27–29]. Furthermore, significantly higher AGE’s levels were reported in patients with osteoporosis, thereby increasing the risk of fractures [28, 30].

Non cross-linking effects are exerted by the binding of AGE’s to the receptor for AGE’s (RAGE). A wide variety of cells express RAGE, and the interaction with AGE’s incites activation of intracellular signalling, gene expression, and production of pro-inflammatory cytokines (such as Interleukin (IL)-6, Tumor Necrosis factor (TNF)-alpha), and free radicals. At the peripheral (tissue) level, these inflammatory processes exhibit powerful proteolytic activity whereby the collagen becomes more vulnerable and tissue elasticity decreases [31, 32]. At the central level (central nervous system), interaction between AGE’s, Amyloid-beta and hyperphosphorylated tau-protein induce microglia and astrocytes to upregulate the production of reactive oxygen species, pro-inflammatory cytokines, and nitric oxide which affects neuronal function [11, 32].

Numerous reviews [16, 33–39] on the relation between AGE’s and motor function have been published, but the majority of these studies are narrative reviews and therefore showing most often an indirect relationship. The negative effect of accumulating AGE’s on the biomechanical properties of peripheral musculoskeletal tissues and the central nervous system (CNS), for example accumulation in specific relevant motor-related brain regions, will plausibly have an impact on motor function. However, evidence regarding this subject is fragmented, and a systematic review on this topic is lacking. Therefore, the aim of this study was to systematically review the literature for the direct relationship between circulating and/or tissue AGE’s and motor function.

## Methods

### Search strategy

The Embase, Pubmed, Cinahl, and Web of science databases were searched from the earliest possible date through January 2016. Terms were employed both as free text words and mapped to subject headings terms with explosion when feasible. We utilized all possible relevant terminology regarding AGE’s and motor function (decline). For detailed information on search terms, see Table 1. The search regarding terms for AGE’s were combined with our search (with AND) regarding terms for motor function (decline). Search filters were established for studies on humans and the English, Dutch, or German language. Bibliographies from identified articles were reviewed for additional references.

**Table 1:** Detailed search terms

Entry	Keywords
AGE’s	“advanced glycosylation end products” (OR) “advanced glycosylation end products receptor (supplementary concept)” (OR) “advanced glycation end product(s)” (OR) “advanced glycation end product(s) receptor” (OR) “non-enzymatic glycation” (OR)“ non-enzymatic glycosylation” (OR) “glycotoxins”.
AND	
Motor function	“musculoskeletal physiological phenomena” (OR) ”muscle fatigue” (OR) “muscle strength” (OR) “muscle rigidity” (OR) “muscle tonus” (OR) “muscle stiffness” (OR) “mechanical stiffness” (OR) “rigidity” (OR) “ physical endurance” (OR) “postural balance” (OR) “balance” (OR) “posture” (OR) “postural control” (OR) “articular range of motion” (OR) “psychomotor performance” (OR) “motor activity” (OR) “motor skills” (OR) “motor skills disorders” (OR) “coordination” (OR) “motor coordination” (OR) “motor control” (OR) “eye-hand coordination” (OR) “fine motor” (OR) “fine motor skills” (OR) “neuromuscular manifestations” (OR) “gait” (OR)“gait disorders” (OR) “walking” (OR) “locomotion” (OR) “falls (accidental)” (OR) “mobility limitation” (OR) “physical mobility” (OR) “physical performance” (OR) “physical activity” (OR) “activities of daily living” (OR) ”motor function” (OR) “motor function decline” (OR) “mobility impairment” (OR) “functional decline” (OR) “motor inhibition” (OR) “inhibition of action” (OR) “interlimb” (OR) “interlimb coordination”.

### Inclusion criteria and outcome variables

Studies were included providing that a direct relationship between measured AGE’s levels and motor function was described. Motor function was defined as any movement or activity through the use of motor neurons in a broad sense (i.e. motor skills, motor control, coordination, locomotion etc.) and/or physical performance (i.e., activities of daily life (ADL), walking, stair climbing, etc.) and/or musculoskeletal function (i.e., muscle strength or stiffness, range of motion etc.). Studies were excluded when merely the effects of AGE’s on tissue level or their relation with specific diseases where described, without any direct outcome on motor function.

### Data extraction and study quality

Data were independently extracted by two reviewers. A standardized data extraction form was exploited to derive the following data from each eligible study: first author, year of publication, study design, study population (age, gender, and co-morbidity); the type of AGE’s (e.g., crosslinking, non-crosslinking, and chemical characteristics); and the outcome of motor function and the strength of the relationship between AGE’s and motor function (e.g., correlation coefficients, risk ratio’s etc.). The methodological quality of each incorporated study was assessed independently by two reviewers. All eligible studies turned out to be observational studies and were assessed with the 12 item EBRO Assessment Tool of Cohort Studies [40]. This tool comprises eight validity questions, one question to decide whether to continue the checklist, two applicability items, and one item for statistical analysis. The two applicability items were disregarded as they merely answer questions to determine if the results are applicable to Dutch healthcare. Therefore, ten criteria remained (Table 2). The included studies were qualified as “good”, “moderate”, or “low”. To qualify as “good”, the study should rate “valid and applicable” and include sufficient statistical analyses (e.g., correction for potential confounders). A “moderate” qualification was assigned when the validity items rated “doubtful” and included sufficient statistical analyses or rated only “valid and applicable” for the validity items. Any other scenario would have given the study a “low” methodological qualification (Table 2). In the event of a discrepancy, the final score was decided by consensus.

**Table 2:** Methodological quality of observation studies with EBRO assessment tool of cohort studies [38]

Item	Semba et al. 2010 [45]	Sun et al. 2012 [46]	Whitson et al. 2014 [47]	Shah et al. 2015 [50]	Dalal et al. 2009 [43]	De La Maza et al. 2008 [49]	Momma et al. 2011 [44]	Tanaka et al. 2015 [48]
1. Are the comparing groups clearly described?	Yes	Yes	Yes	Yes	Yes	Yes	Yes	Yes
2. Can risk of bias sufficiently be excluded?	Yes	Yes	Yes	Yes	Yes	Yes	Yes	No
3. Is the exposure clearly described and is the method for assessing the exposure adequate?	Yes	Yes	Yes	Yes	Yes	Yes	Yes	Yes
4. Is outcome clearly described and is the method for assessing the outcome adequate?	Yes	Yes	Yes	Yes	Yes	Yes	Yes	Yes
5. Has exposure outcome been blinded?	Yes	Yes	Yes	Yes	Yes	Yes	Yes	Yes
6. Is there sufficient long follow-up?	Not Applicable	Yes	Yes	Not Applicable	Not Applicable	Not Applicable	Not Applicable	Not Applicable
7. Can selective loss-to-follow-up sufficiently be excluded?	Not Applicable	Yes	Yes	Not Applicable	Not Applicable	Not Applicable	Not Applicable	Not Applicable
8. Have important confounders or prognostic factors been identified and are they taken into account in the design or analysis?	Yes	Yes	Yes	No	Yes	No	Yes	Yes
9. Are the study results valid and applicable?	Yes	Yes	Yes	Yes	Yes	Yes	Yes	Yes
10. Correction for potential confounders: Odds ratio (OR), Relative Risk (RR), Absolute Risk Reduction (ARR), Mean Difference (MD), Hazard Ratio (HR)	Yes	Yes	Yes	No	Yes	No	Yes	Yes
Overall quality score	Good	Good	Good	Moderate	Good	Moderate	Good	Good

To be eligible for further statistical analysis, the study results should rate “valid and applicable” or “doubtful” on question 9. To qualify “valid and applicable” a longitudinal study had to score “Yes” on the 2 follow up questions (6 and 7) and more than 4 times a “yes” on the remaining validity items (1 t/m5 and 8). A “doubtful” qualification was given when 4 times a “yes” was scored on the remaining validity items (1 t/m5 and 8). When a study scored “yes” less than 4 times on the remaining items, further analysis with the checklist was cancelled. When the study had a cross-sectional design the two questions regarding follow up (6 and 7) were disregarded

#### Statistics

Risk ratios (RR), Odds ratio’s (OR), Hazard ratio’s (HR), correlation coefficients, and group differences (mean difference and 95 % confidence interval) were presented as reported in the studies. The subjects’ ages were presented as reported in the studies or in means and standard deviations (SD) that were calculated from the available data. If association coefficients were not available, effects sizes (ES) [41] were calculated to estimate the magnitude of the effect of AGE’s on specific outcomes when the mean difference and standard deviation of compared groups were available or could be retrieved from confidence intervals.

The following formula were used:

$$ SD = \surd\ \left( number\  of\  subjects\right)\ *\ \left[ upper\  limit - lower\  limit\right]/\left(2*\ 1.96\right) $$

$$ ES=\left[ mean\ 1\ \hbox{--}\ mean\ 2\right]/SD\  pooled,\  where\ SD\  pooled = \surd\ \left[\left(S{D_1}^2 + S{D_2}^2\right)/2\right] $$

Cohen’s benchmarks were used to indicate small (ES = 0.20), medium (ES = 0.50), and large (ES = 0.80) effects size [42]. Due to the heterogeneity across the studies, meta-analysis was not performed.

## Results

### Search results

**Fig. 1:**
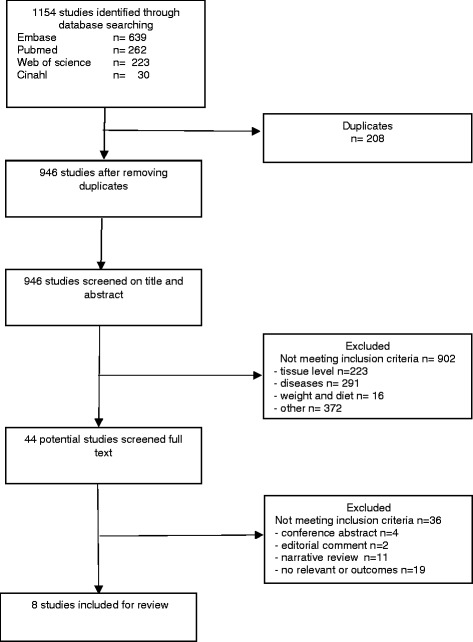
Flow Chart of selection process

The literature search revealed 1154 hits. After eliminating 208 duplicates and after an initial screening of titles and abstracts, 902 studies were excluded. Primary reason for exclusion were that the studies were describing an (indirect) effect only on tissue level (*n* = 223), were disease related (*n* = 291) or in the field of diet and weight (*n* = 16), all without outcome on movement, activity, performance and or function. The remaining excluded studies (*n* = 372) did not meet the inclusion criteria in general. A total of 44 full-text articles were retrieved for further analyses. Examination of the available reference lists of these 44 studies did not reveal additional studies. From the 44 potentially eligible studies, 36 studies were excluded because no relevant outcomes were described (*n* = 19), were narrative reviews (*n* = 11) or were conference abstracts (*n* = 4) and editorial comments (*n* = 2). Finally, eight studies were included for this review. A flowchart of the selection process is presented in Fig. 1.

### Study quality

The study quality of six studies was scored as “good” [43–48]. A moderate score was assigned to two studies [49, 50] because potential confounders were not taken into account in the analysis (Table 2).

### Data extraction

#### Study design

Of the eight included studies, six had a cross-sectional design [43–45, 48–50], one had a longitudinal design [46], and one study employed a mixed longitudinal and cross-sectional design [47].

#### AGE’s type

The chemical characteristics of the reported AGE’s was described in six studies [43, 45–49]. Carboxymethyl-Lysine (CML) was quantified in five studies [43, 45–47, 49] and Pentosidine in one study [48]. In two studies the chemical characterisation of the AGE’s were not specified, yet measured indirectly by skin fluorescence [44, 50]. For obtaining the AGE’s levels, blood samples were used in five studies [43, 45–48]; tissue samples in one study [49]; and the non-invasive AGE reader in two studies [44, 50].

#### Motor function outcomes

The outcomes for motor function from the eight retrieved studies were categorized into three categories;

1. Physical performance and functioning: walking abilities [45, 46], activities of daily living (ADL) [47] and upper extremity function [50];
2. Musculoskeletal: muscle strength, power and mass [43, 44, 48, 49];
3. Physical frailty: in accordance with the Fried criteria [8] and defined as having three or more of the following characteristics: low physical activity, exhaustion, slow walking speed, decreased muscle strength, and unintentional weight loss [47].

Study characteristics and an overview of the main findings and statistics from the studies are provided in Table 3.

**Table 3:** Study characteristics

First author/year of publication	Design	Population n =, age, gender and co-morbidity	AGE/RAGE Circulating/Tissue levels, Tissue type	Musculoskeletal outcome	Physical performance and functioning outcome	Main findings and Outcome statistics:
Semba et al. 2010 [45]	Cross-sectional	Community dwelling older adults *N* = 944 (416 M, 528 F), aged 75 ± 7 years	Circulating plasma CML (cut-off value for high level = 424 ng/mL)	-	Walking speed (cut-off value for slow walking <0.79 m/s)	Risk for slow walking speed with high AGE’s level: OR = 1.60 (95 % CI:1.02–2.52)*^c^ OR = 1.87 (95 % CI:1.15–3.04)*^c^ (+ adjusted for DM2)
Sun et al. 2012 [46]	Longitudinal observational study with 30 month follow-up	Moderately to severe disabled community dwelling F, *N* = 394, aged 76 ± 8 years	Circulating serum CML (cut-off value for high level = 689.1 ng/mL)	-	Walking disability (inability or slow speed) (cut-off value for slow walking <0.4 m/s)	Walking disability risk with high AGE’s level: HR = 1.68 (95 % CI: 1.11–2.52)* HR = 1.63 (95 % CI: 1.06–2.49)*^c^(1) HR = 1.56 (95 % CI: 1.04–2.36)*^c^(2) HR = 1.54 (95 % CI: 1.04–2.29)*^c^(3) HR = 1.46 (95 % CI: 0.95–2.23) NS.^c^(4)
Whitson et al., 2014 [47]	Longitudinal observational study with 14 year follow-up for ADL disability. Cross-sectional for physical frailty components	Community dwelling older adults *N* = 3373 (1344 M, 2029 F) aged 78.1 ± 4.8 years	Circulating serum CML (cut-off value for high level = 620 ng/mL)	ADL disability Physical frailty ≥3 of following characteristics: 1. Low handgrip strength 2. Unintentional weight loss 3. Low physical activity 4. Exhaustion 5. Slow walking speed		ADL disability risk with high AGE’s level: HR = 1.08 (95 % CI: 1.03–1.13)*^b^ HR = 1.10 (95 % CI: 1.05–1.15)*^c^ HR = 1.05 (95 % CI: 1.01–1.11)*^c^ (+ adjusted for arthritis, cognition, kidney disease) Physical frailty risk with high AGE’s level in men: HR = 1.30 (95 % CI: 1.14–1.48)*^b^ HR = 1.24 (95 % CI: 1.07–1.45)*^c^ HR = 1.10 (95 % CI: 0.92–1.32) NS.^c^ (+ adjusted for arthritis, cognition, kidney disease) Physical frailty risk with high AGE’s level in women: HR = 1.06 (95 % CI: 0.91–1.23) NS.^b^ HR = 1.06 (95 % CI: 0.90–1.24) NS.^c^ HR = 0.91 (95 % CI: 0.77–1.07) NS.^c^ (+ adjusted for arthritis, cognition, kidney disease)
Shah et al. 2015 [50]	Cross-sectional	DM2 patients *N* = 26 (13 M, 13 F) aged 64,5 ± 6,8 Non DM *N* = 26 (13 M, 13 F) aged 64,2 ± 5,8	AGE-type not defined (skin tissue auto fluorescent Crosslinking AGE’s)	Shoulder flexor strength	1. Upper extremity disability 2. Upper extremity function	Correlation flexor strength and AGE’s level: R = 0,07 NS Correlation Upper extremity disability and AGE’s level: R = 0,51* Correlation Upper extremity function and AGE’s level: − humerothoracic elevation R = −0.44* − glenohumeral external rotation R = −0.32 NS
Dalal et al. 2009 [43]	Cross- sectional	Moderately to severe disabled community dwelling F, *N* = 559, aged 76 ± 8 years	Circulating serum CML (cut-off value for high level = 0.68 mg/mL)	Handgrip strength	-	Group difference high vs. low AGE’s level: 18.2 (6.4) kg. vs. 20.1 (6.2) kg., Beta −1.88 (SE = 0.65)* 18.6 kg vs. 20.0 kg, Beta −1.31 (SE = 0.61)*^c^
De La Maza et al. 2008 [49]	Cross-sectional	Healthy M, *N* = 21 − Weight maintainers, *N* = 10, − aged 41 ± 4 years − Weight gainers, *N* = 7, aged 42 ± 5 years − Elderly, *N* = 4, aged 67 ± 2 years	Skeletal muscle tissue CML/RAGE	Handgrip strength	-	Correlation grip strength and AGE’s level: R = −0,54*
Momma et al. 2011 [44]	Cross-sectional	Healthy M, aged 46 years (37–56)^a^ - Grip strength analysis, *N* = 232 - Leg extension analysis, *N* = 138	AGE-type not defined (skin tissue auto fluorescent Crosslinking AGE’s) (cut-off value for high level = 2.09–4.44 AF)	1. Handgrip strength 2. Leg extension power	-	Group difference high vs low AGE’s level: 1. Muscle (handgrip)strength: 41.7 (95 % CI: 40.3- 43.1) kg. vs. 44.5 (95 % CI: 43.2- 45.9) kg.*^c^ ES = 0.45 2. Muscle (leg extension) power: 16.0 (95 % CI: 14.9- 17.1) W/kg. vs. 17.8 (95 % CI: 16.6- 19.1) W/kg.*^c^ ES = 0.44
Tanaka et al. 2015 [48]	Cross-sectional	DM2 patients, F, *N* = 133, aged 66,8 ± 9,5 years	Circulating serum Pentosidine	1. Upper extremity muscle mass 2. Lower extremity muscle mass 3. Relative skeletal muscle mass index		Association UMM and AGE’s level: R = −0.11 NS Beta −0.11 NS Association LMM and AGE’s level: R = −0.21* Beta −0.18* Association RSMI and AGE’s level: R = −0.18* Beta −0.27*

*M* Male, *F* Female, *yrs* years, *AGE* Advanced Glycation End product, *RAGE* Receptor for Advanced Glycation End products, *CML* Carboxy-methyl-Lysine, *DM2* Diabetes Mellitus type *2*

^a^Median (interquartile range), *ES* Effect Size, *OR* Odds Ratio, *HR* Hazard Risk

^b^multivariate adjusted for demographics

^c^idem + multivariate adjusted for potential confounders. *NS* Not significant. (1) All women with missing data had multiple simulations to impute walking disability status prior to death. (2) Inverse probability weighting method. (3) All women with missing data treated as developing walking disability prior to death. (4) All women with missing data treated as censored, that is, no walking disability prior to death, *UMM* Upper extremity Muscle Mass, *LMM* Lower extremity Muscle Mass, *RSMI* Relative Skeletal Muscle Mass Index

^*^ *P* < 0,05

### Relationship of AGE’s with physical performance and functioning

#### Walking abilities

Walking and the relationship with Carboxymethyl-Lysine (CML) was studied in two studies [45, 46]. In the cross-sectional study by Semba et al. [45], walking speed was assessed in a representative sample of community dwelling elderly (*n* = 944). Slow walking speed was defined as the slowest quintile of walking speed which was <0.79 m/s. Plasma CML was divided into quartiles with the cut-off at the highest quartile established at 424 ng/mL. The study demonstrated that, after adjusting for covariates (age, education, smoking, cognition status, depression, and chronic disease), the subjects in the highest quartile of plasma CML were at greater risk of a slow walking speed (OR = 1.60, 95 % CI: 1.02–2.52, *P* = 0.04) compared to those in the lower three quartiles of plasma CML. After an exclusion of subjects with diabetes and adjusting for the same covariates, the study showed that participants in the highest quartile of plasma CML were at a higher risk for slow walking speed, (OR = 1.87, 95 % CI 1.15–3.04, *P* = 0.01). In the longitudinal study by Sun et al. [46], walking speed and serum CML were measured in moderately to severely disabled community dwelling female subjects (*n* = 394). Walking disability was defined as the inability to walk or a walking speed of <0.4 m/s. Cut-off values according to plasma CML quartiles were 452.6, 558.8 and 689.1 ng/mL. The study showed that subjects in the highest quartile of CML were at a greater risk for developing a walking disability (HR = 1.68, 95 % CI: 1.11–2.52, *P* = 0.03). Multivariable hazard models for different missing data scenario’s (for details, see Table 3) and adjusting for health characteristics (age, congestive heart failure, peripheral artery disease, diabetes mellitus type 2 (DM2), and renal insufficiency) revealed a significantly higher risk for women in the highest quartile of CML to develop a walking disability (HR’s between 1.46 and 1.63, all *p* < 0.05).

#### Activities of Daily Living (ADL)

The ability to perform ADL activities and the longitudinal relation with CML in older persons was described in the study by Whitson et al. [47]. Serum CML was measured in participants in the Cardiovascular Health study (*n* = 3373) and was divided into quintiles with the cut-off for the two highest quintiles established at 620 ng/mL. ADL disability was defined as difficulty (yes or no) in any of six ADL activities such as walking around the home, getting out of bed/chair, dressing, bathing, eating, and toileting and was assessed at 6- or 12- month intervals for 14 years. AGE levels were compared between the groups with and without difficulty on the specific ADL activities. After adjusting for potential confounders (demographics, BMI, DM2, alcohol consumption, smoking status, total cholesterol, albumin, weight change, hypertension, heart disease, stroke, and claudication), a high level CML at baseline was associated with 10 % (95 % CI 5 %–15 %) higher incidence of a first ADL difficulty with a hazard ratio (HR) of 1.10 (95 % CI:1.05, 1.15), *p* < 0,01) per standard deviation (225 ng/mL CML). After further adjusting for arthritis, cognition, and kidney function, an increase in CML was associated with a 5 % (95 % CI 1 %–10 %) higher incidence of disability, HR of 1.05 (95 % CI:1.01–1.11, *P* = 0.03) per standard deviation (225 ng/mL CML) in both males and females.

#### Upper extremity function

Upper extremity function and the relation with AGE’s was studied by Shah et al. [50] in groups with diabetes mellitus type 2 (DM2) (*n* = 26) and without DM2 (*n* = 26). Upper extremity disability was measured using the Disability of the Arm, Shoulder and Hand (DASH) self-report questionnaire. The DASH has 30 questions, including disability and pain. The scores were calculated for a range between 0 and 100 %, where a higher number indicates more impairments. Skin tissue fluorescent AGE’s levels were assessed utilizing an AGE reader. The AGE reader auto-fluorescence (AF) was correlated to the DASH scores (r = 0.51, *P* = 0,009) indicating that higher AGE levels correlate with increased disability of arm, shoulder or hand. They also studied the three-dimensional humerothoracic and glenohumeral joint motion and found that AGE reader levels were negatively correlated to humerothoracic elevation (r = −0.44, *P* = 0.024), but not correlated to glenohumeral external rotation (r = −0.32, *P* = 0.13).

### Relationship of AGE’s with Musculoskeletal outcome

#### Muscle strength and power

The contribution of AGE’s on the muscle properties strength and power was described in four studies [43, 44, 49, 50]. Dalal et al. [43] examined handgrip strength and circulating CML concentrations in moderately to severely disabled, older females (*n* = 559). The quartile cut-offs for serum CML were 0.45, 0.55, and 0.68 mg/mL. Women in the highest quartile of CML experienced decreased mean grip strength compared with women in the lower three quartiles, 18.6 kg. vs. 20.0 kg, respectively, with a Beta of −1.88 (SE = 0.65, *P* = 0.004). After adjusting for covariates (age, race, BMI, cognition, depression, and DM2), a difference was reported of 18.2 (6.4) kg. vs. 20.1 (6.2) kg, respectively, with a Beta of −1.31 (SE = 0.61, *P* = 0.03). The evidence of tissue CML/RAGE in skeletal muscle (internal abdominal oblique) biopsies of healthy subjects (*n* = 21) differing in age and weight stability was studied in the cross-sectional study by de la Maza et al. [49]. The subjects (*n* = 21) were divided into a group of self-reported weight maintainers (WM)(*n* = 10); self-reported weight gainers (WG) (*n* = 7); and elderly (E) (*n* = 4). The study ascertained that handgrip strength was diminished in subjects with an increase of CML/RAGE in muscle tissue and reported a negative correlation (r = −0.54, *p* < 0.05) between tissue CML and handgrip strength.

The relationship between AGE’s and grip strength and leg extension power was described in the study by Momma et al. [44]. In their study, healthy adult males (*n* = 370) were divided into two groups, i.e., one group (*n* = 232) for analysing grip strength and another group (*n* = 138) for leg extension strength. Skin tissue fluorescent AGE’s levels were assessed utilizing an AGE reader. The tertile cut-off ranges for the AGE reader auto-fluorescence (AF) were 1.28–1.84, 1.84–2.09 and 2.09–4.44. After adjusting for potential confounders (age, BMI, physical activity, smoking/drinking status, depression, education, occupation, total energy consumption, metabolic syndrome, DM2, kidney disease, and dietary intake of vitamin C), it was discovered that grip strength was 6.3 % less for those in the highest tertile than for subjects in the lowest tertile of skin AF (*P* = 0.01). Leg extension power was 10 % lower for those in the highest tertile than for the lowest tertile of AF (*P* = 0.04).

Shoulder flexor muscle strength and the relation with AGE’s was described in the study by Shah et al. [50] in groups with DM2 (*n* = 26) and without DM2 (*n* = 26). Skin tissue fluorescent AGE’s were assessed utilizing an AGE reader. The study revealed that AGE reader auto-fluorescence (AF) was not related to shoulder flexor muscle strength (r = 0.07, *P* = 0.7).

#### Muscle mass

Loss of muscle mass and the relation with serum levels of Pentosidine was studied in the study by Tanaka et al. [48] In this study among postmenopausal women with DM2 (*n* = 133) upper and lower extremity muscle mass was measured by Dual-energy x-ray absorptiometry and also the relative skeletal muscle mass index (RSMI = appendicular skeletal muscle mass/height^2^) was calculated. They showed that serum Pentosidine levels were negatively correlated with muscle mass of the lower extremity (r = −0.21, *P* = 0.0017) and RSMI (r = −0.18, *P* = 0.039), but not with the muscle mass of the upper extremity (r = −0.11, *P* = 0.219). After correction for covariates (age, DM2 duration, serum creatinine, glycosylated haemoglobin (HbA1c) and insulin-like growth factor-1 (IGF-1)) they reported that serum Pentosidine levels were associated with RSMI with a Beta of −0.27 (*P* = 0.008) and muscle mass of the lower extremity with a Beta of −0.18 (*P* = 0.071).

### Relationship of AGE’s with physical frailty

Physical frailty in relation to CML was reported by Whitson et al. [47]. Frailty was based on the five criteria previously described by Fried el al. [8]: less physical activity, self-reported exhaustion, decreased grip strength, slow walking speed, and unintentional weight loss. Participants with three or more criteria were classified as physically frail. In this cross-sectional study serum, CML was measured in older, healthy participants (*n* = 3373). Self-reported exhaustion was identified by evaluation of two statements of the Centre for Epidemiological Studies Depression Scale (CES-D) and was regarded positive if at least one condition was evident for three or more days during the previous week. The decreased physical activity criterion was positive if physical activity per week was <383 kcal/week for men and <270 kcal/week for women. Slow walking speed and inadequate grip strength were defined as the lowest 20 % of the population. Finally, unintentional weight loss was defined as unintentional loss of 4.5 kg in the year prior to the current evaluation or unintentional weight loss of at least 5 % of the previous year’s body weight.(7) AGE levels were compared between groups with or without presence of the specific frailty components. Mean CML levels were significantly higher in men for low physical activity (*p* = 0.007), exhaustion (*p* < 0.001) and decreased grip strength (*p* = 0.04), resulting in a significant association with the physical frailty phenotype (OR = 1.30, 95 % CI:1.14–1.48, *p* < 0.001). After adjusting for potential confounders (demographics, BMI, DM2, alcohol consumption, smoking status, total cholesterol, albumin, weight change, hypertension, heart disease, stroke, and claudication), a significant association remained (OR = 1.24, 95 % CI:1.07–1.45, *p* = 0,04). The associations lost significance after further adjustment for arthritis, cognition, and kidney function. No significant associations between CML and low physical activity, exhaustion, and grip strength were determined for females. Furthermore, in men and women with a slow walking speed and unintentional weight loss, CML levels were higher, however, these differences in CML levels were not significant.

## Discussion

The results of this systematic review indicate that higher levels of AGE’s are independently related to declined walking abilities, inferior ADL, decreased muscle properties (strength, power, mass) and increased physical frailty.

The available literature on musculoskeletal outcomes [43, 44, 48, 49] support the hypothesis that high AGE’s levels are associated with a decline in muscle function. However, the correlations, Beta coefficients and calculated effect sizes indicate only a moderate relationship. It is known that AGE’s can affect muscle function through a variety of pathways. In fact, AGE’s can alter the biomechanical properties of muscle tissue, increasing stiffness and reducing elasticity through cross-linking and upregulated inflammation by RAGE binding and endothelial dysfunction in the intra-muscular microcirculation [43–46, 49, 51]. This is also consistent with studies on sarcopenia in which decreased muscle mass and strength is explained by an overall increase in inflammatory burden [6]. Examining the studies in this review that report decline in walking abilities, it is suggested by the authors that this decline is also attributed to the effects of AGE’s on muscle tissue, thereby impairing muscle function [45, 46]. It has been considered that impaired muscle function - through AGE’s-induced muscle damage – can contribute to decline in walking abilities and ADL and can also contribute to physical frailty.

It remains ambiguous to what extend ADL involving the upper extremity are affected by high AGE’s level. One small study [50] reports a relation with upper extremity disability, but further we could not identify which ADL are affected most by AGE’s and if at all are related to upper extremity function. Thereby in a large study [47] the reported increased risk in inferior ADL is described as the association between baseline AGE’s level and the time to first difficulty on any of the six self-reported ADL items and, although significant, the risk is low (HR = 1.10), therefore the practical importance is unclear.

Furthermore our review shows that the decline of handgrip strength is positively associated with higher AGE levels in three studies [43, 44, 49]. Interestingly, in the upper extremity shoulder muscles no association with high AGE levels is found in two studies [48, 50]. The underlying mechanism for this difference in the upper extremity remains unclear and further research is necessary on this topic.

We identified only 8 studies due to strict inclusion criteria for several reasons; 1) We restricted the inclusion to individual studies that reports a direct relationship between AGE’s and functioning which enabled us to calculate effect sizes, association coefficients etc. for direct comparison of the strength of the association. 2) To increase internal validity we were specifically interested in motor functioning so we excluded papers measuring solely an effect on tissue levels. 3) Also we did not include papers that only cover a specific disease (such as Diabetes) to increase external validity. As mentioned previously, AGE’s have an effect on different types of human tissues [24, 26, 31, 32]. It must be considered that other AGE’s-induced effects, such as effects on the nervous system, ligaments, tendons or joint capsules, could contribute to the decline of physical performance and functions. Future research should, therefore, explore other possible underlying AGE’s related pathways, not only on the pathophysiological changes on the tissue level, but also with a direct relation on motor function.

The current systematic review shows that the relation between AGE’s and motor function is not as strong as most narrative reviews suggest. Although the quality of the included studies was good to moderate, they employed a cross-sectional or observational design, therefore, a causal relationship cannot be inferred. A meta-analysis was not appropriate because of the heterogeneity of the studies.

It is known that, in addition to cognitive decline, accompanying decline in motor function is frequently reported in patients with dementia [52] and it is suggested that AGE’s formation may explain many biochemical and neuropathological changes in the most common cause of dementia [13]. However, the results from this review provide no conclusive evidence for a peripheral or a central level effect of AGE’s on motor function in Alzheimer’s disease or other types of dementia. We were unable to locate any study describing the effect of AGE’s on the CNS with a direct relation on motor function. It remains unclear if AGE’s accumulate in specific relevant motor-related brain regions, having their effect on the complex inter-relationship between the distributed motor networks within the CNS as well as with the musculoskeletal structures for generating movement. Future research is required to determine the contribution of AGE’s accumulation on the CNS with a direct relation on the decline in motor function.

It is important to realise that, in this review, decline in motor function was primarily associated with elevated CML levels. Association with circulating CML was determined in four studies [43, 45–47], and a relation with tissue CML was found in one study [49]. One study reported an association with Pentosidine [48] and two other studies with non-specified skin tissue fluorescent AGE’s [44, 50]. It is suggested that fluorescent and non-fluorescent AGE’s such as CML behave similarly and fluorescence may be employed as a marker for the total skin tissue AGE’s pool [53]. Although CML is a dominant AGE in blood circulation and correlates with other AGE’s [14], it is possible that the association between AGE’s and motor function outcome could be different if crosslinking AGE’s such as Pentosidine were assessed.

In five studies, only females [43, 46, 48] or males [44, 49] were included, therefore, the reported decline in motor function with elevated AGE’s cannot necessarily be extrapolated to the other gender. Previous longitudinal studies conducted in a large cohort suggest that the relation between serum AGE’s and health elevated levels of serum AGE’s were associated with all-cause mortality, cardiovascular disease, and coronary heart disease mortality only in women [54, 55]. In contradiction, Whitson et al. [47] found a significant cross-sectional association between CML and physical activity, exhaustion, and muscle strength as components of physical frailty only among men and not among women. A possible explanation suggested by Whitson et al. is that aged cohorts may be subject to gender-specific survivor bias. They state that the relative significance of CML mediated factors in determining the risk of death is greatest for females prior to menopause but greater for men in advanced ages. If AGE’s have more effect on women, higher-risk women may then be unlikely to participate in studies that measure CML late in life due to the negative health outcomes [47]. On the other hand, they did find an association with men and women in their longitudinal study on ADL disability. In a comparable cohort, Semba et al. [45] reported a strong association between men and women and do not report any significant gender differences. Therefore, this raises an additional question of whether or not the effect of AGE’s could be gender specific. Future research should, therefore, include gender-based differences in the effects of AGE’s.

The vast majority of participants included in this review were elderly people older than 64 years (*n* = 5433). Interestingly, in two studies [44, 49], the participants were middle-aged between 37 and 56 years (*n* = 387). This indicates that the negative effect of AGE’s on motor function already begins during midlife and, as AGE’s levels increase with ageing, could be an important factor in age related decline in motor function. A high AGE’s level, as a biomarker, therefore, could predict a decline in motor function later in life. This could also imply that preventive interventions should start as soon as possible as part of healthy ageing. In accordance with the results of this review, it would be interesting to investigate whether motor function can be improved by reducing AGE’s levels. Intensive glycaemic control may be a method to decrease AGE’s formation. CML levels correlate to dietary consumption [14], therefore, dietary intake is a possible factor that can be influenced. It is suggested that, in order to lower daily AGE’s intake, foods rich in sugar and fat and those prepared by frying or grilling should be avoided [14, 56]. However, evidence of the harmful effects of long-term exposure to dietary AGE’s is still inconclusive [15]. Improvements of glycaemic control by regular physical exercise could also attenuate the formation and accumulation of AGE’s [11, 21, 57]. Elevated AGE’s levels might be an indication to initiate (early) treatment such as dietary advice, muscle strengthening exercises, and functional training to maintain physical functions. However, literature regarding the effects of physical exercise on AGE’s formation is minimal, and the optimal exercise modalities remain ambiguous [21]. Further research should focus on whether dietary modifications, exercise programs, or medication to reduce AGE’s levels can prevent or counter decline in motor function.

## Conclusion

This review indicates that decline in motor function is independently associated with high AGE’s levels. Further longitudinal observational and controlled trial studies are necessary to investigate a causal relationship, and to what extent, high AGE’s levels are a contributing risk factor and potential biomarker for decline in motor function as a component of the ageing process.

## Abbreviations

AD: Alzheimer’s disease
ADL: activities of daily life
AF: auto-fluorescence
AGE’s: Advanced Glycation End products
BMI: body mass index
CES-D: Centre for Epidemiological Studies Depression Scale
CML: carboximethyl-lysine
CNS: central nervous system
DASH: disability of the arm shoulder and hand
DM: diabetes mellitus
DM2: diabetes mellitus type 2
EF: effects size
HbA1c: glycosylated haemoglobin
HR: hazard ratio
IGF-1: insulin-like growth factor-1
IL-6: interleukin-6
OR: odds ratio
RAGE: receptor for advanced glycation end products
RR: risk ratio
RSMI: relative skeletal muscle mass index
SD: standard deviation
TNF: tumor necrosis factor
WG: weight gainers
WM: weight maintainers
